# Secondary metabolism of *Microcystis*: current understanding and recent advances in unlocking genomic and chemical diversity

**DOI:** 10.1128/aem.01634-25

**Published:** 2026-01-05

**Authors:** Colleen E. Yancey, Lauren N. Hart, Gregory J. Dick

**Affiliations:** 1 Department of Earth and Environmental Science, University of Michigan 1259, Ann Arbor, Michigan, USA; 2 Program in Chemical Biology, University of Michigan 1259, Ann Arbor, Michigan, USA; 3 Life Sciences Institute, University of Michigan 123743, Ann Arbor, Michigan, USA; 4 Cooperative Institute for Great Lakes Research (CIGLR), School for Environment and Sustainability, University of Michigan 1259, Ann Arbor, Michigan, USA; The Pennsylvania State University, University Park, Pennsylvania, USA

**Keywords:** metagenomics, metabolomics, harmful algal blooms, multi-omics, secondary metabolites, *Microcystis*

## Abstract

The cyanobacterial genus *Microcystis* is globally distributed and known for its ability to produce microcystins, a structurally diverse group of cyanotoxins. However, the biosynthetic capacity of *Microcystis* is vast; its diverse genomes contain a variety of biosynthetic gene clusters (BGCs) encoding the synthesis of metabolites that may be toxic, have important ecological function, or have applications for biotechnology or drug discovery. Recent studies illustrate that these BGCs vary significantly across *Microcystis* strains, can be highly expressed in environmental conditions, and may play key roles in cellular physiology, grazer deterrence, and microbial interactions. However, many of these BGCs and metabolites remain poorly characterized or completely uncharacterized, having been identified only through genome sequencing or mass spectrometry, respectively, leaving no knowledge of their structure, bioactivity, or physiological or ecological functions. Here, we synthesize the current body of knowledge regarding the secondary metabolism of *Microcystis* in terms of genetic and chemical diversity, potential drivers of synthesis, and physiological and ecological functions. This review highlights the need for further research to characterize the largely unexplored genetic and chemical diversity of *Microcystis* in communities in the environment and discusses the challenges and opportunities of integrating high-throughput multiomic approaches to link uncharacterized gene clusters with their corresponding metabolites. *Microcystis* will continue to be a rich source for secondary metabolite research as its genetic and chemical potential likely plays a critical role in the persistence and observed dynamics of harmful algal blooms and may harbor uncharacterized toxins and metabolites.

## INTRODUCTION

*Microcystis* spp. are among the most common bloom-forming cyanobacteria responsible for cyanobacterial harmful algal blooms (cyanoHABs) that degrade freshwater systems around the world (1). These cyanoHABs have been observed on every continent except Antarctica (2), and the presence of *Microcystis* in brackish and coastal waters (3, 4) underscores its widespread distribution. Within blooms, *Microcystis* can achieve dominance and persist seasonally (5–8), and it encodes the capacity to produce a breadth of secondary metabolites that have toxic properties (2, 9–11). These blooms can lead to several negative consequences including toxin production (2, 10, 12, 13), shifts in community composition (9, 14), and hypoxia (15). Freshwater blooms dominated by *Microcystis* threaten access to clean drinking water and recreation through the production of toxins and taste and odor compounds (11, 14, 16). Similarly, in coastal and estuarine systems, there have been reports of disruption in fish production in aquaculture and the accumulation of toxins in surface water, aquatic life, and human nasal passages (3, 4, 17, 18). Together, these findings underscore the broad distribution and impact *Microcystis* imposes on aquatic ecosystems.

CyanoHABs are primarily driven by eutrophication from anthropogenic sources of nutrients. Phosphorus (P) loading has been documented to drive bloom biomass and is the main target of management practices (19, 20). Continued nitrogen (N) loading, which is not officially managed in most North American freshwater systems, may favor toxic strains as many secondary metabolites with toxic properties are N-rich (21, 22). As a result of eutrophication and increased environmental variability since the Industrial Revolution, the intensity and frequency of cyanobacterial blooms are increasing in freshwater and marine systems, disproportionately to other taxa of phytoplankton (1). Models predict that by the year 2090, there will be 18–39 days of intense harmful algal bloom growth versus the average 7 days currently experienced in temperate systems (23). Studies aimed at addressing the impacts of climate change on bloom severity have shown that elevated temperature and carbon dioxide levels will not only increase *Microcystis* biomass but also microcystin content per cell (24, 25). Intensifying environmental variability and nutrient loading emphasize the need to better understand the consequences of persistent cyanobacterial biomass in aquatic systems, especially prolific toxin producers such as *Microcystis*.

Studying *Microcystis* genomes, and their biosynthetic potential, is challenging due to the high levels of diversity observed among strains. Further challenges arise in taxonomic identification and species delineation as a result of the complex nature of *Microcystis* genomes (2, 26) and their varied cell size and colony morphology (27). Due to their genetic complexity and lack of clear species and sub-species organization via phylogenomic approaches (28, 29), *Microcystis* blooms likely comprise ecologically distinct strains adapted to variable environments (28). *Microcystis* genomes have highly variable gene content across strains and thus have a large pangenome (26, 30), with a high degree of horizontal gene transfer (26, 29, 31). High levels of plasticity are also evidenced by extensive regions of repeat sequences within genomes and low synteny among strains, which may be a strategy used to adapt to shifting environments (26, 28, 30). It has also been suggested that the *Microcystis* pangenome is truly globally distributed (30). Such diverse genetic substructure among strains (28, 29) provides even more potential for biosynthetic diversity.

**Fig 1:**
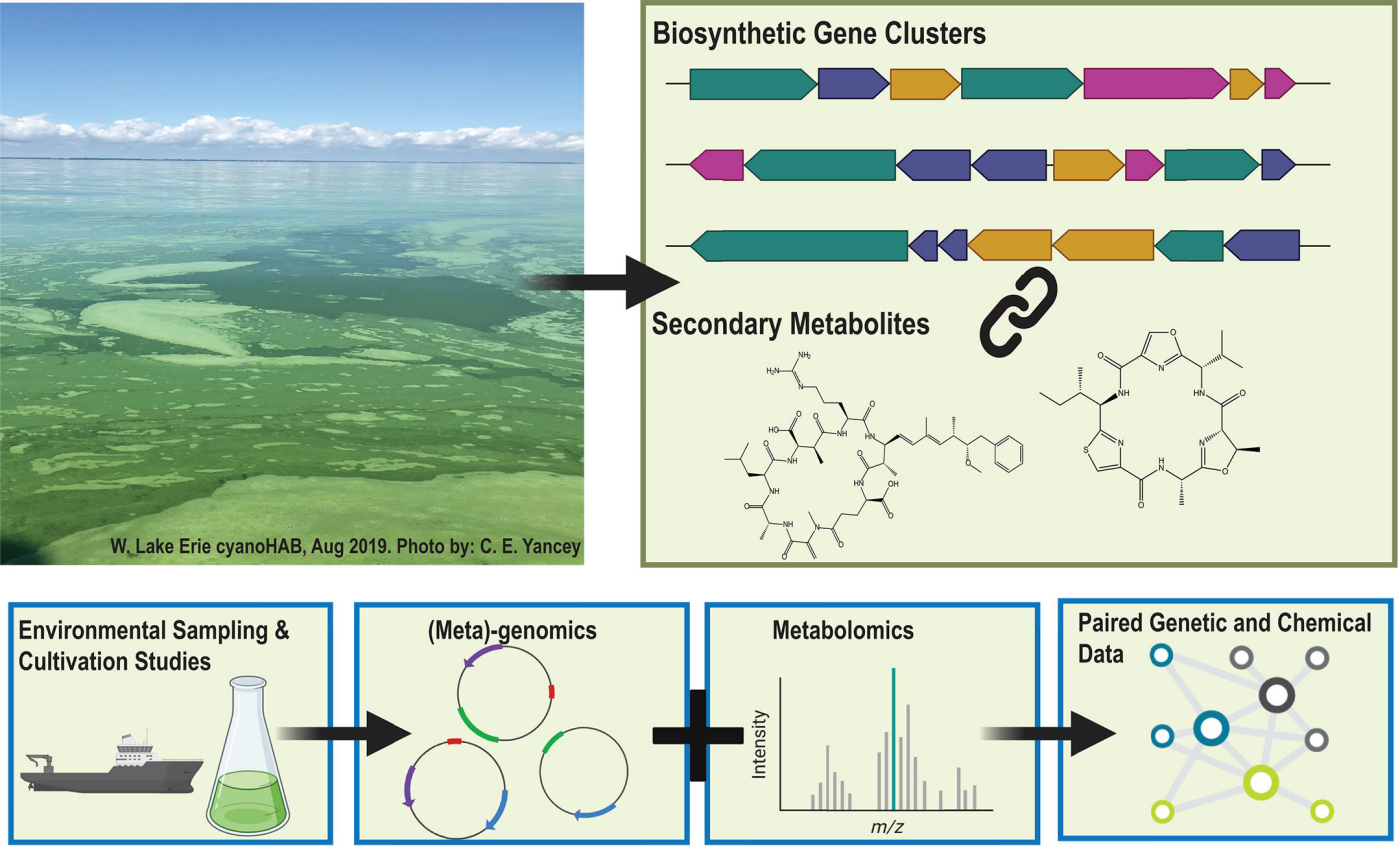
Schematic overview of workflows used in *Microcystis* secondary metabolism studies. Lake water collected from visible algal scums can be used for metagenomic and metabolomic analyses. These methods enable the identification of biosynthetic gene clusters and secondary metabolites for further study and characterization.

In general, cyanobacteria are a rich source of unique, toxic, and complex secondary metabolites (5, 8, 32). Several classes of “cyanotoxins” produced by various genera have been described previously and are monitored in water sources around the world (33–35). To date, many studies and reviews have focused on secondary metabolites produced broadly by cyanobacteria, largely based on studies of cultures, or focus solely on the hepatotoxin microcystin, which currently dominates cyanobacteria secondary metabolism research and literature (5, 6, 33, 36, 37). Harke et al. (2) reviewed canonical toxins produced by *Microcystis*, focusing on microcystins, but they did not address the many other cyanopeptides produced by *Microcystis*. While some studies have addressed the variable genome content of biogenetic clusters (BGCs) within *Microcystis* (28, 29, 38, 39), and others have identified and characterized the chemical structures of specific compounds (40–45), to our knowledge, the field currently lacks a comprehensive review of the expansive chemical and genetic diversity that defines *Microcystis* secondary metabolism. This review synthesizes the state of knowledge regarding *Microcystis*-derived metabolites, identifies areas for continued research, and addresses how improvements in omics technology may advance the field (Fig. 1). We hope that it will serve as a valuable resource for researchers as *Microcystis*-dominated cyanoHABs expand and intensify globally along with climate change.

## GENOMIC INSIGHTS INTO DIVERSITY OF SECONDARY METABOLITES

**Fig 2:**
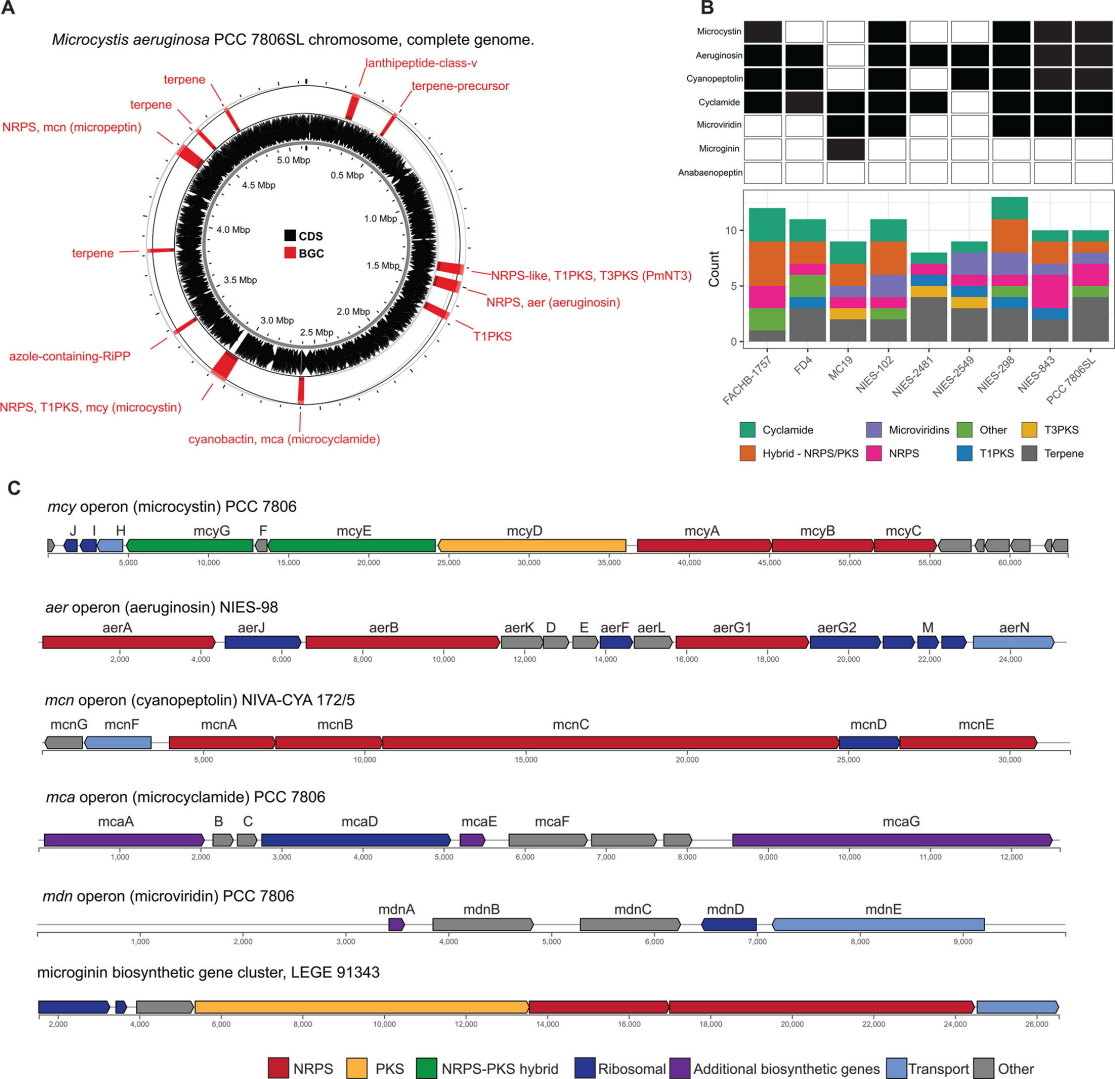
Overview of BGCs found in *Microcystis* spp. genomes. (**A**) Genomic map of the complete genome from *Microcystis aeruginosa* PCC 7806 (NCBI accession: NZ_CP020771.1). Both characterized and uncharacterized BGCs are depicted and labeled in red. Coding DNA sequences (CDSs) are shown in black. The genomic map was generated with Proksee. (**B**) Summary of BGCs from *Microcystis* genomes with finished status (level 6) from IMG (accessed July 2025). The top panel shows the presence or absence of gene clusters that encode characterized secondary metabolites (black indicates presence). The bottom panel shows the count and types of both characterized and uncharacterized biosynthetic gene clusters, highlighting vast genetic diversity and the need for continued exploration in well-characterized isolates. (**C**) Select examples of common BGCs found in *Microcystis*. Identified CDSs are labeled for each cluster where available. Gene schematics were modified from the MiBIG repository (accessed July 2025).

Studies of *Microcystis* cultures revealed their high diversity of secondary metabolites and the BGCs encoding their synthesis (5, 38). BGCs make up about 2%–7% of *Microcystis* genomes, and they are often distributed genome-wide (Fig. 2A) (38). Horizontal gene transfer likely plays a role in BGC acquisition (26, 31, 38), although some clusters, such as the *mcy* operon that encodes microcystin, have been shown to have ancient origins, and their distribution among genera is primarily due to gene loss (46). BGCs are also thought to be dynamic, with tightly regulated controls on transcription and frequent rearrangements in gene order (38, 39, 47). It is likely that multifaceted combinations of biotic and abiotic factors have contributed to the evolution of the BGCs observed today in *Microcystis* genomes (28, 35), yet much remains unknown about their variation on a species or subspecies level and why these metabolites are being synthesized from a functional standpoint. Currently, there are 13 linked BGCs and metabolites deposited on the Minimum Information about a Biosynthetic Gene Cluster (MiBIG) database (48) that are known to be produced by *Microcystis* (accessed July 2025) (Table 1). All entries describe gene clusters that encode cyanopeptides either synthesized via nonribosomal peptide synthetase (NRPS), hybrid NRPS/polyketide synthase (PKS), or ribosomal pathways.

**TABLE 1:** Summary of identified metabolites and BGCs encoded in *Microcystis* genomes and deposited on the MiBIG database (accessed July 2025)^*a*^

Strain(s)	Secondary metabolite(s) identified	Biosynthetic mechanism	Biosynthetic genes	Bioactivity	Reference
PCC 7806	Microcyclamide 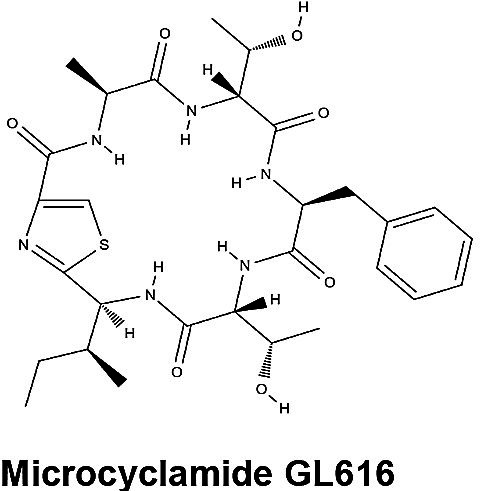	Ribosomal	*mcaA-mcaG*	Moderate cytotoxicity against P388 murine leukemia cells, cardiotoxicity and lethality of zebrafish (LC50 = 43 µg/mL), and potential grazer deterrent	(49, 50)
	Microcystin 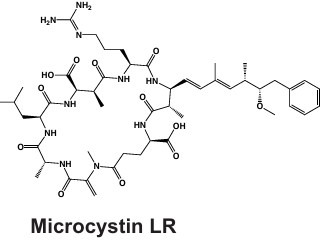	NRPS, PKS, and hybrid NRPS- PKS	*mcyA-mcyJ*	Hepatotoxin via protein phosphatase PP1A and PP2A inhibition	(51, 52)
K-139, *Microcystis* sp. NIVA-CYA 172/5	Microcpeptin/cyanopeptolin 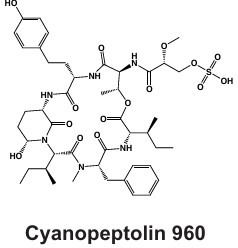	NRPS	*mcnA-mcnG*	Eukaryotic trypsin inhibition, crustacean cytotoxicity/lethality	(53–56)
NIES-298, MRC	Microviridin B, Microviridin J 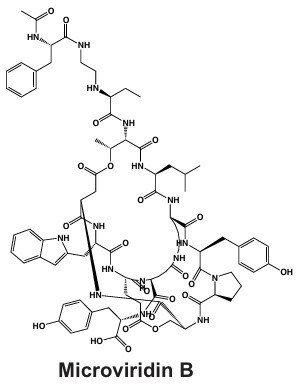	Ribosomal	*mdnA-mdnD*	Protease inhibition including trypsin, elastase, and chymotrypsin	(49, 57, 58)
NIES-98	Aeruginosin 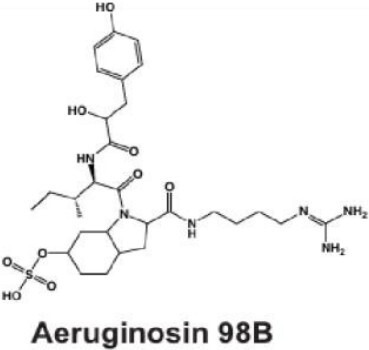	NRPS	*aerA-aerN*	Trypsin and thrombin inhibition	(59–62)
NIES-298	Aerucyclamide 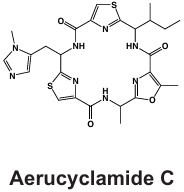	Ribosomal	*mcaA-mcG*	Antiparasitic activity against *P. falciparum* and *T. brucei* and potential grazer deterrent	(49, 63)
PCC 9432	Aeruginosamide 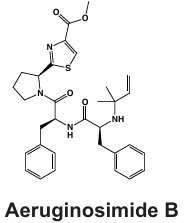	Ribosomal	MICCA_2630 002- MICCA_2630 007	Cytotoxicity against human breast cancer cells and mild inhibitory activity against human cytochrome P450	(64, 65)
PCC 7005	Piricyclamide 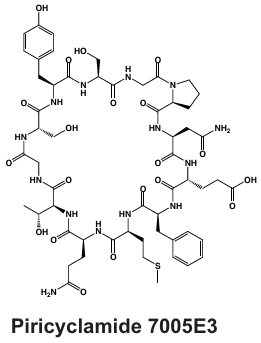	Ribosomal	*pirA-pirG*	Potential grazer deterrent	(47)
NIES-87	Kasumigamide 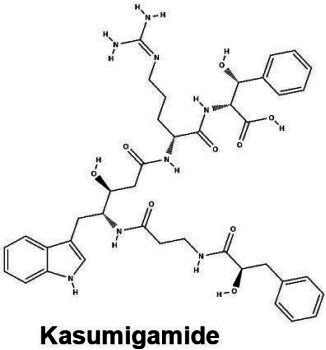	NRPS-PKS	*makasAmakasD*	Growth inhibition of *C. neglecta* (MIC = 2 µg/mL)	(66)
LEGE 91341	Microginin 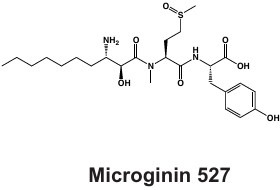	NRPS-PKS	IQ234_09865- IQ234_09895	Angiotensin-converting enzyme inhibition and aminopeptidase inhibition	(67)
Western Lake Erie Culture Collection Isolates	Anabaenopeptin 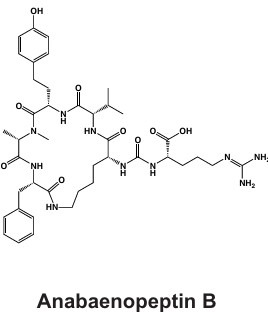	NRPS	*apnA-E*	Protease inhibition including serine proteases and protein phosphatases	(68)

^*a*^ Due to emerging detection in *Microcystis* genomes and blooms, anabaenopeptins are also included. For each secondary metabolite discussed, a congener is also displayed. Chemical structures were obtained from PubChem (https://pubchem.ncbi.nlm.nih.gov/).

Genomic content of BGCs across strains within *Microcystis* subclades tends to be similar but not identical (28, 29, 69). It is likely each strain or species of *Microcystis* contains a co-evolved, tailored arsenal of secondary metabolites that are fine-tuned to the specific conditions of their environment (28). However, the presence of a gene cluster does not guarantee biosynthesis as these clusters may be transcriptionally regulated and deactivated through transposition or recombination (38, 39, 47), which are commonly observed in *Microcystis* (2, 26). Biosynthesis in natural systems is also likely dependent on several abiotic and biotic factors such as the availability of substrates, C:N ratios within water bodies, and interaction with competitors and grazers (70, 71). As a result, it is important to use genome mining as a blueprint for biosynthetic *potential*, but approaches such as metatranscriptomics and chemical profiling (e.g., mass spectrometry) are critical to determine which secondary metabolites are being actively biosynthesized.

## CHARACTERIZED SECONDARY METABOLITES

*Microcystis* can produce a wide range of secondary metabolites, with varying function, toxicity, and chemical structure. However, understanding of the biosynthetic processes underlying *Microcystis* secondary metabolite production remains limited, beyond a few well-characterized cyanopeptides (2, 36). The following sections summarize what is currently understood about *Microcystis* secondary metabolites, the genes that encode them, and some of the key knowledge gaps that remain.

### Microcystin

First identified in 1959 as the “fast death factor” (72), microcystin (MC) and the related hazards surrounding this toxin have been at the forefront of *Microcystis* secondary metabolism research (36). Several reviews focus on MCs (37, 73, 74); here, we briefly summarize key aspects, recent advances, and remaining questions. MCs are efficient eukaryotic protein phosphatase 1 and 2A inhibitors that can lead to illness including liver damage, and in extreme cases, death (75, 76). Within the last 30 years, MC intoxication has been reported in humans (76, 77), sheep (78), and other mammals and birds (79). MCs have been responsible for drinking water crises in the United States (14) and China (11), when levels of microcystin exceed the World Health Organization (WHO) guidelines for maximum concentration in drinking water (1 µg/L).

Structurally, MCs are cyclic heptapeptides (Table 1) that contain the unusual (2S,3S,8S,9S)−3-amino-9-methoxy-2,6,8-trimethyl-10-phenyldeca-4,6-dienoic acid (Adda) domain (74, 80), which has become an essential marker in detection assays (81). The structure was first determined in 1984 (80), and 279 congeners have since been characterized (74). MCs contain highly variable X and Z amino acid positions that can contain leucine, arginine, tyrosine, and other amino acids (73, 74), which can greatly impact the toxicity of the congener produced (73, 82). For example, MC-LR, one of the most common forms of MCs, is over 100 times more toxic than MC-RR (83). Continued MC research aims to discover new congeners, understand their chemical ecology (36, 74), and uncover the determinants and roles of congener diversity. While the gene sequence influences which MC congeners are synthesized (84, 85), amino acid availability (86), relaxed substrate binding specificity (84), the availability and type of nitrogen (71), as well as carbon: nitrogen (C:N) ratios (87) can also influence the final chemical structure.

MCs are synthesized nonribosomally via a multienzyme complex that contains NRPS, PKS, and hybrid PKS-NRPS enzymes. In *Microcystis*, the cluster contains 10 *mcy* genes encoding biosynthesis and putative tailoring and transport enzymes and is controlled by a bidirectional promoter between *mcyA* and *D* (Fig. 2C) (51). The *mcy* BGC is dynamic, with frequent recombination and point mutations, and these shifts in genetic substructure can impact congener production (84, 85, 88, 89). The *mcy* genes *A, B,* and *C* are the most hypervariable in sequence structure (84). Recently, a novel partial *mcy* genotype, in which only *mcyB* and *C* and a truncated *mcyA* are present, and *mcy* genes *D-J* are absent, was detected in western Lake Erie and found to be abundant and transcriptionally active (89). Further work suggested that this partial operon encodes a tetrapeptide that shows signs of bioactivity, eliciting mild elevation of some markers of hepatotoxicity and inflammation in human liver epithelial cell lines (90). Although more work is needed to assess the toxicity of this molecule, these findings highlight the capacity for genetic rearrangement of *mcy* genes to generate novel metabolites, as well as our limited understanding of diversity and depth of *Microcystis* secondary metabolite biosynthesis despite intensive study of MCs for over 50 years.

**Fig 3:**
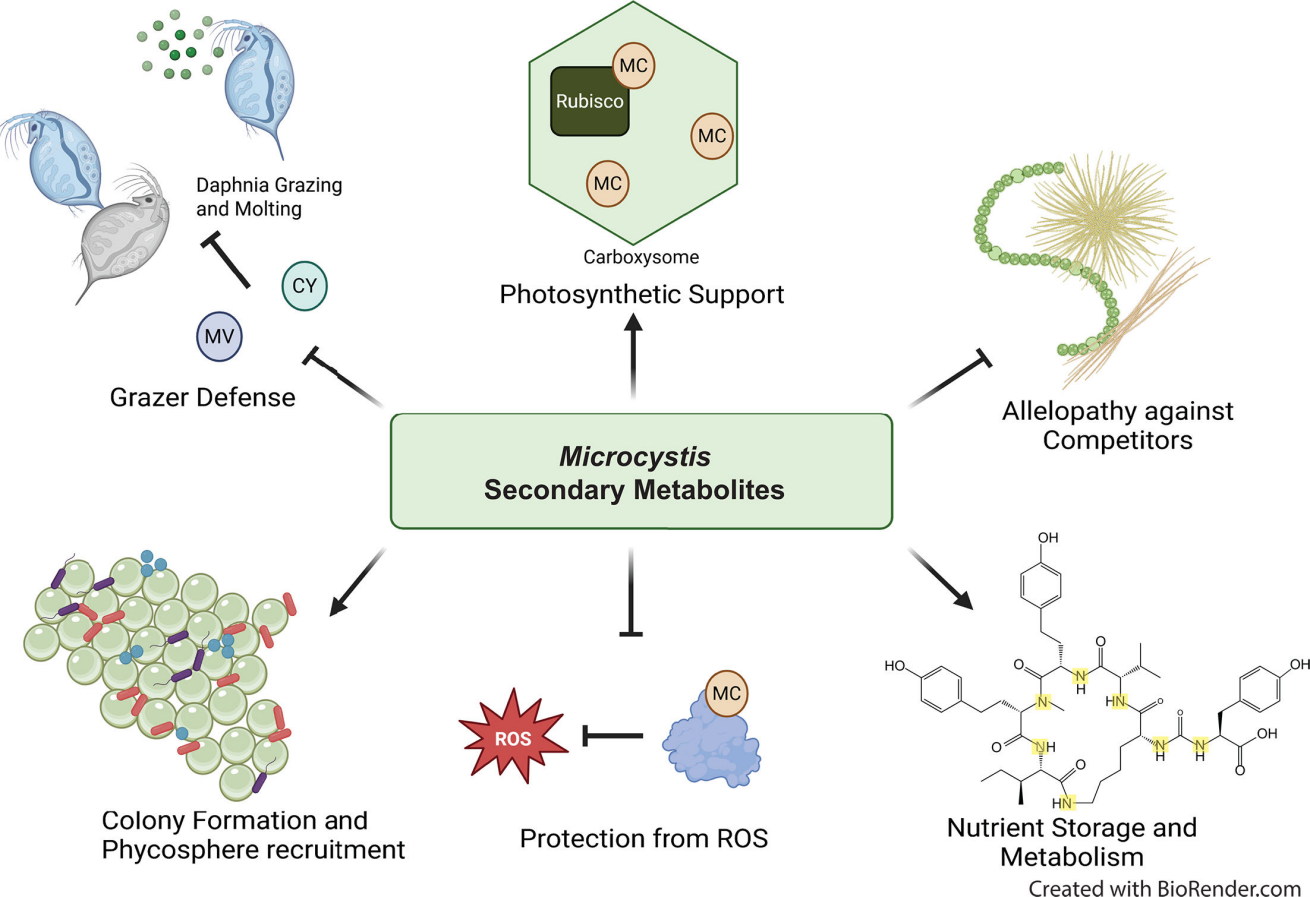
Proposed ecological functions of synthesized *Microcystis* secondary metabolites. Secondary metabolites produced by *Microcystis* likely have multifaceted functionality and may support (clockwise from top left) the following: defense from grazers, photosynthetic function, colony formation and phycosphere recruitment, photosynthetic machinery, competition, nitrogen storage and metabolism, protection from ROS, and/or recruitment and selection of the phycosphere microbiome. For example, cyanopeptides such as cyclamides (CY) and microviridins (MV) may deter grazers and inhibit *Daphnia* molting (70, 93). Microcystins (MC) and other cyanopeptides are involved in photosynthesis and carbon assimilation (91) (reviewed in (91), allelopathy (94), nitrogen storage and metabolism (95), binding proteins to protect from ROS (96), and shaping the phycosphere microbiome (97). N-rich Anabaenapeptins (bottom right) may also play a role in N storage and metabolism.

Despite the well-recognized global importance and impacts on human and environmental health of MCs, their functional role(s) in *Microcystis* physiology and in natural communities remains elusive. Proposed roles of MCs include benthic survival and recruitment, iron acquisition, nutrient metabolism and storage, grazer defense, colony formation, allelopathy, quorum sensing, oxidative stress protection, and photosynthesis (Fig. 3) and are summarized in Section 5 and elsewhere (37, 91). Given that many of these hypotheses have experimental support, it seems likely that MCs have a multifaceted functionality. Understanding this functionality would be valuable from a basic scientific perspective, and it could also inform predictive models, strategies, and policies to mitigate bloom toxicity as it relates to MC production (21, 92).

### Characterized secondary metabolites beyond microcystin

While over 90% of research on cyanobacterial secondary metabolites has been focused on MCs (36, 98), many other metabolites with diverse chemical structures are produced and may contribute to bloom toxicity and/or affect food web dynamics and remain understudied (99–101). Multiple cyanopeptides are often present in cyanoHABs, and recent studies show that these cocktails may have synergistic toxicological effects on aquatic organisms and humans (102, 103).

#### Aeruginosins

Aeruginosins are linear tetrapeptides (Table 1) that inhibit trypsin activity and are encoded in NRPS BGCs (Fig. 2C) (59, 60, 104). Like many classes of *Microcystis* secondary metabolites, aeruginosins contain unusual moieties including 4-hydroxyphenyllactic acid (Hpla) and 2-carboxy-6-hydroxyoctahydroindole (Choi) (59, 104). Multiple congeners of this metabolite have been identified (40, 105, 106), including those that are brominated or chlorinated (107). While the Hpla and Choi moieties tend to be conserved, there is flexibility within amino acids at the second position, which may contribute to the observed chemical variation (104, 108, 109).

Some of the diversity observed in aeruginosin congeners may be due to the highly varied and dynamic nature of the aeruginosin biosynthesis genes in the *aer* operon (60, 61). Core genes responsible for the bulk of NRPS synthesis are conserved in most *aer* operons, but accessory or tailoring genes are more varied in both sequence structure and presence or absence (38, 109). For example, the presence and sequence variation of genes *aerJ*, *aerG2,* and *aerM* may be responsible for the synthesis of chlorinated isoforms (38), while *aerK* appears to be essential for biosynthesis by *Microcystis,* but not *Planktothrix* spp (109). Aeruginosin class-related secondary metabolites including aeruginoside are also produced by *Microcystis* (64), while others, such as spumigin and pseudospumigin, are produced by other cyanobacterial taxa (109–111). It remains unclear whether *Microcystis* can produce these related metabolites as well. While this class of secondary metabolites is highly diverse and has strong inhibitory properties against trypsin and thrombin (62), the functional role of aeruginosins in natural communities is not well understood (53, 103, 112, 113).

#### Cyanopeptolins

Cyanopeptolins are peptide lactones that were first characterized in the *Microcystis aeruginosa* isolate PCC 7806 (114). These depsipeptides contain lactone rings, a 3-amino-6-hydroxy-2-piperidone (Ahp) residue, and n-hexanoic acid moieties (Table 1) (114, 115). Cyanopeptolins can occur in similar concentrations (nanomolar) as MCs in surface freshwater and can cause inhibitory effects on eukaryotic organisms via trypsin inhibition (36, 115). Micropeptins, such as micropeptin K139, are structurally related to cyanopeptolins and can be synthesized by *Microcystis* as well (116, 117). Several congeners inhibit crustacean activity in concentrations as low as the picomolar range (56), raising questions regarding their threats to organism and ecosystem health.

The NRPS biosynthetic gene cluster that encodes for cyanopeptolins is highly varied, even within the same genus (35, 54, 55). Flexibility in the *mcn* gene cluster (Fig. 2C) is so great that operons may lack entire genes and still synthesize complete cyanopeptolin congeners (38). Cyanopeptolins are synthesized by multiple cyanobacteria genera including *Microcystis, Planktothrix*, and *Anabaena*, which may contribute to its chemical diversity. Phylogenetic analysis has revealed the gene cluster that encodes this metabolite has independently evolved in these three taxa and that *mcnA-F* encodes its production in *Microcystis* (54, 116). The halogenase gene *mcnD* is sporadically distributed across *mcn* BGCs and has been linked to the production of chlorinated cyanopeptolin variants (60). While gene clusters, structural characteristics, and inhibitory properties have been studied from cultured isolates, cyanopeptolins are largely understudied but increasingly detected in natural communities (39, 99).

#### Cyclamides: Piricyclamides, Aerucyclamides, Microcyclamides, and Aeruginosamides

Another common and diverse group of secondary metabolites produced by *Microcystis* are the “cyclamides,” which are ribosomally synthesized macrocyclic molecules within the cyanobactin class (8, 118). Being one of the largest groups of secondary metabolites found in *Microcystis* and other cyanobacteria, cyclamides are classified together based on their ribosomal and post-translationally modified biosynthetic pathways (RiPPs) (119). Characterized cyclamides produced by *Microcystis* include piricyclamides, aerucylcamides, microcyclamides, and aeruginosamides (Table 1) (47, 49, 63, 64). These metabolites may have various functional groups in their chemical structures: prenylations, grenylations, and disulfide bridges, which are observed in piricyclamides (47); cyclic hexapeptide structures as seen in microcyclamides (120); and oxazole and thiazole rings observed in the side chains of aerucyclamide compounds (63).

The gene clusters that encode for cyclamide metabolites (Fig. 2C) are also highly varied and can be inactivated in culture by insertion elements (47). Some evidence suggests that synthesized products are used in grazer defense as their concentration was observed to increase in *Microcystis* cells consumed by *Daphnia* in grazer experiments. Cells containing cyclamides were actively exported out of the *Daphnia* body, without any further degradation, suggesting these compounds may be a filter feeding deterrent (70). Additional studies have demonstrated selective antiparasitic activity of aerucyclamides (63). These metabolites have been detected simultaneously in culture in combination with other known cyanopeptides (94), highlighting the need to investigate the synergistic effects of co-occurring *Microcystis* secondary metabolites in natural bodies of water. This highly diverse class has high potential for drug discovery due to its potential versatility in biotechnology.

#### Microviridins

Microviridins are also ribosomally synthesized metabolites (Fig. 2C), but they are unique as they contain unusual tricyclic structures and several ester bonds (Table 1) (41, 121). These metabolites are believed to be the first tricyclic compounds isolated from nature and have tyrosinase inhibitory effects (41). After ribosomal synthesis, microviridins are tailored by ATP-grasp ligases and transporter peptidases to finalize their chemical structure (122). Chemical variation observed in microviridins may be in part due to gene variation (8, 38). Precursor peptide genes such as *mdnA* lack conservation across *Microcystis* strains and may contribute to the chemical diversity of this class (123). Absence of *mdnD* (Fig. 2C) could account for microviridins lacking N-acetylation (38). Microviridin congeners have a range of cytotoxic effects from highly lethal to undetectable (8). For example, microviridin J is a strong inhibitor against *Daphnia* molting (93), while microviridin B (Table 1), which demonstrates weaker protease inhibition, may be more suitable for biomedical application (122).

#### Microginins

*Microcystis* also produces microginins, which are linear peptides that inhibit a variety of peptidases (Table 1) (124, 125). The BGC encoding for microginins is a hybrid NRPS/PKS cluster (Fig. 2C) (126) and was recently confirmed to be present in *Microcystis* along with the production of 12 novel congeners (67). These peptides can range greatly in size from three to six amino acids long and tend to derive from decanoic acid (125–127). Microginins are highly diverse, with as many as 50 congeners existing in a single bloom (127). Microginin congeners may be produced in tandem with microcystin (128). Recently, microginins have received more attention due to their angiotensin-converting enzyme (ACE) inhibitory activity and potential application in pharmaceuticals (129). This cluster or class of metabolites remains poorly understood, but should be considered in future studies and screenings due to its bioactivity and recent identification in natural *Microcystis* blooms and culture (39, 67, 127).

#### Anabaenopeptins

Along with many other cyanobacterial genera, *Microcystis* can produce anabaenopeptins (Table 1), cyclic hexapeptides with nanomolar inhibitory effects on mammalian carboxypeptidases, via the NRPS BGC *apn* (130–132). This class of cyanopeptides is the second-most studied after microcystins, with a rapidly growing body of literature covering the chemical diversity and ecology of the metabolites within it (98). This BGC is not verified in the MIBiG database, although many studies have confirmed the presence of anabaenopeptin BGC and its synthesis product in unialgal *Microcystis* cultures (69, 133), with over 124 congeners verified from this metabolite class across cyanobacterial genera (68). This BGC was likely acquired from a horizontal gene cluster from *Planktothrix* spp. and may contribute to bloom dynamics in multialgal communities (134). A recent report showing that anabaenapeptins are often present at concentrations higher than microcystins in the western basin of Lake Erie (135) highlights the need for a better understanding of their impacts on human and ecosystem health.

## UNCHARACTERIZED SECONDARY METABOLITES

Genomes of *Microcystis* display an overwhelming number of BGCs that have not yet been linked with a known metabolite, potentially signaling a vast array of undiscovered biosynthetic diversity (Fig. 2A and B) (69). This was identified as a challenge as far back as 2013, with the identification of “orphan BGCs”—those without a known biosynthetic product – in sequenced *Microcystis* genomes (26). The enormity of this knowledge gap has come into view more clearly over the years with accumulation of more (meta)genomic data. The challenge of linking already characterized or newly discovered compounds with their corresponding BGCs in a high-throughput manner can be facilitated with integrated analysis of paired genomic and metabolomic data (136).

A total of 58 high-quality genomes belonging to the genus *Microcystis* are deposited in the Joint Genome Institute (JGI) supported Integrated Microbial Genomes and Microbiomes (IMG/MER) database (https://img.jgi.doe.gov/, accessed July 2025). While most of these genomes come from well-studied culture isolates, their biosynthetic repertoire remains coarsely resolved. From these genomes, 13 BGCs have been characterized and deposited onto the MiBIG database (Table 1, accessed July 2025; Fig. 2). Gene annotation may provide hints about metabolites of interest; however, pairing both metabolomic and genomic data is essential to understand the synthesis and structure of these secondary metabolites. Since *Microcystis* genomes tend to have about 10 to 15 BGCs per genome, and many of these have not been linked to a product (Fig. 2A and C), continued research is needed to directly link strains, BGCs, and chemical structures.

While exploring the extent and diversity of “cyanopeptides” is important (8, 34), the expansive wealth of BGCs encoding various PKS, terpene, and ribosomal pathways should not be ignored. Compounds synthesized via PKS pathways in *Microcystis* remain largely uncharacterized. Several novel PKS-BGCs identified from western Lake Erie metagenomes are transcriptionally active (39), suggesting the synthesis of molecules with polyketide properties. Other works have shown that *Microcystis* may be a rich source for type III PKSs, which are highly understudied in cyanobacteria (69, 133, 137). In Lake Erie cyanoHABs, these same BGCs can be among the most abundant and highly expressed (39), underscoring the need to understand their physiological and ecological functions.

## ENVIRONMENTAL DRIVERS

Anthropogenic eutrophication and environmental variability contribute to the persistence, intensification, and spread of cyanoHAB events globally (1, 12, 138, 139). Phosphorus (P) inputs have long been identified as a driving force contributing to cyanoHAB formation (20). However, nitrogen (N) has also been identified as a limiting or co-limiting nutrient, ultimately altering cyanoHAB composition, intensity, and toxicity (10, 12, 140), suggesting that dual nutrient management would be beneficial in many systems (141, 142). Other factors that must be considered in predicting future cyanoHABs include increasing temperatures and atmospheric carbon dioxide concentrations, as well as decreases in dissolved oxygen within the water column (138). However, growing evidence suggests that nutrient availability, specifically the building blocks of secondary metabolites, which tend to be N-rich compounds (Table 2), may have the greatest impact on *Microcystis* secondary metabolism.

**TABLE 2:** C:N ratios for various known *Microcystis* secondary metabolites, highlighting that several are considered N-rich^*a*^

Compound	Molecular formula	C count	N count	C:N
Aeruginosin	C_36_H_55_N_6_O_9_	36	6	6
Anabaenopeptin 908	C_45_H_68_N_10_O_10_	45	10	4.5
Anacyclamide A10	C_49_H_72_N_12_O_14_	49	12	4.08
Cyanopeptolin	C_40_H_63_N_9_O_14_S_1_	40	9	4.44
Microcyclamide	C_26_H_30_N_8_O_4_S_2_	26	8	3.25
Microcystin L,R	C_49_H_74_N_10_O_12_	49	10	4.9
Microginin	C_32_H_52_N_4_O_7_	32	4	8
Microviridin B	C_84_H_106_N_16_O_24_	84	16	5.25
Piricyclamide	C_56_H_78_N_10_O_16_S	56	10	5.6

^*a*^ Chemical formulas were obtained from the Minimum Information about a Biosynthetic Gene Cluster (MiBiG) repository (https://mibig.secondarymetabolites.org/).

The contribution of N to *Microcystis-*dominated cyanoHABs has recently received greater attention. Elevated concentrations of N, which is not currently restricted by either the United States or Canada within the Great Lakes, but is managed in waters in the European Union (143), can not only alter the species composition but may also favor “toxic” strains. N availability, and its stoichiometric relationship to carbon (C) and P, affects both the amount of microcystins produced and the relative abundance of congeners (22, 144, 145). Some modeling efforts also suggest that planned P reductions will not only decrease biomass but also alleviate N-limitation, thus enhancing the production of N-rich secondary metabolites such as microcystins (21, 92). It is possible that similar trends may be observed for other N-rich secondary metabolites such as aeruginosins and microginins (Table 2), although current models focus solely on MCs.

Differential congener production may also be influenced by amino acid availability, intracellular C:N ratios, and substrate availability resulting from metabolic exchange with other microorganisms (71, 86, 87, 146). These controls on metabolite production are imperative to understand as different congeners have varying potency and toxicity, and unmanaged N may alter abiotic conditions that select for more N-rich metabolites. While many studies focus on microcystin production, N is also required to synthesize other N-rich cyanopeptides, and exogenous N availability is expected to influence their cellular quotas as well (142). Thus, future work should be expanded to include a greater range of N-rich secondary metabolites produced by *Microcystis* (Table 2). Another emerging area of research is understanding how the *Microcystis* microbiome contributes to nitrogen processing and uptake, which can influence cyanotoxin production (147).

## PROPOSED FUNCTIONAL ROLES OF SYNTHESIZED SECONDARY METABOLITES

Limited evidence speaks to the functional roles of *Microcystis*-derived metabolites *in situ*. Most hypotheses are derived from culture experiments, and most work on this topic has focused on MCs, for which there is still no consensus (37). Several competing hypotheses exist regarding drivers of production (Fig. 3), and it is possible that secondary metabolites may have multifunctional roles, especially concerning microbial interactions. *In vitro* studies have suggested *Microcystis*-derived metabolites are allelopathic in nature and aid in achieving dominance through inter- and intra-species competition (94, 148). Some studies suggest *Microcystis* secondary metabolites may be used in grazer defense against multiple organisms including *Daphnia* (70) and copepods (149), although other work suggests increased grazing does not stimulate the upregulation of putative grazer defense metabolite synthesis (150). Conversely, it has been suggested these metabolites are not antagonistic in nature, but rather serve as an aid in recruitment of “helper” bacteria within the phycosphere (97, 151). More targeted studies are needed to definitively ascertain how *Microcystis*’s secondary metabolites mediate microbial interactions.

A growing body of work also suggests that *Microcystis* secondary metabolites are produced to aid in intracellular processes rather than communication or allelopathy within natural community assemblages. MCs bind proteins, including RubisCO, providing protection from reactive oxygen species during rapid growth or high light conditions (Fig. 3) (152–155). MCs may also serve as protective agents from ROS during cold temperature acclimatization (156). Since several *Microcystis* secondary metabolites are N-rich (Table 2), they may play a role in metabolism and storage of vital nutrients. For example, the production of MCs is dependent on the N concentration (140) and may be regulated by *ntcA*, a global nitrogen regulator, which binds the *mcy* operon promoter and inhibits transcription during N-depleted conditions (157). Non-MC-producing strains of *Microcystis* require a greater accumulation of proteins involved in N metabolism, suggesting MCs play an important role in N storage in MC-producing strains (95). This research on intracellular roles of MCs should be expanded to explore the intracellular functions of other *Microcystis* secondary metabolites, especially given evidence for linkages and interchangeability between *Microcystis* secondary metabolites (94) and if these compounds may be functionally redundant but more favorable under different environmental conditions.

## ADVANCES IN OMICS TECHNOLOGIES ENABLE A NEW ERA OF SECONDARY METABOLITE RESEARCH

### Advancements

The significant reduction in cost of DNA sequencing has been essential to uncover the extensive intraspecies diversity of *Microcystis* both from cultured isolates and natural populations (28, 89). Third-generation sequencing platforms such as PacBio (158) and Oxford Nanopore (159) are steadily improving the quality of microbial genomes. For example, long-read sequencing recently aided in the completion of closed cyanobacteria genomes belonging to the *Aphanizomenon*, *Dolichospermum*, and *Anabaena* (ADA) clade, which are cyanobacteria with complex genomes similar to *Microcystis*, from environmental sources (160). On the IMG/MER database (accessed July 2025), only nine *Microcystis* genomes are denoted as “Finished.” Of these nine genomes, seven were completed using PacBio sequencing, and all nine were completed on cultured isolates (Fig. 2B), reflecting the challenge of assembling complete genomes with short-read sequencing due to the repeat-rich and heterogeneous nature of *Microcystis* genomes. Future sequencing efforts of *Microcystis* genomes should continue to implement long-read or proximity ligation sequencing (161) within environmental samples to identify cryptic BGCs that may encode novel compounds not observed in culture.

The field of metabolomics is also rapidly expanding due to improvements and accessibility in data processing and analysis tools. The most common method used, due to the ability to sensitively detect large varieties of metabolites, is liquid chromatography mass spectrometry (LC-MS) (162). Tandem mass spectrometry (MS/MS), which is widely applied to environmental samples, has made it possible to identify and distinguish more compounds with secondary fragmentation and feature analysis (163, 164). While these approaches are suitable for novel compound discovery and initial feature characterization, they have also been used to detect known *Microcystis* secondary metabolites with the aid of standards, achieving detection and quantification in less than 24 hours (165).

Recent efforts in developing tools and repositories for collaborative and accessible research have opened new avenues for secondary metabolite research (136). BGC mining software such as antiSMASH (166) and PRISM (167) have rapidly improved over the years and are continually refined and updated through expansive collaborative efforts and machine learning approaches. Data repositories such as the Minimum Information about a Biosynthetic Gene Cluster (MiBiG) (48), the Global Natural Products Social Molecular Networking (GNPS) (168), and the Natural Products Atlas (NPAtlas) (169) provide a wealth of genomic and metabolite data—both of novel and known compounds.

### Opportunities

A major opportunity for secondary metabolite research is the integration of *in situ* ‘omic data sets, i.e., metagenomic, metatranscriptomic, and metabolomic data, into a comprehensive picture of what *Microcystis* is producing and why in natural systems. Many publicly available ‘omics data sets currently contain only a single ‘omic approach and lack metadata of environmental or experimental conditions, making determination of drivers and mechanisms of production challenging. Integrated multiomic data and paired ancillary environmental data are necessary to identify and determine functions of secondary metabolites but are challenging and may require multiple modeling and networking approaches (170, 171). Employing “multi-omic” data set integration with basic modeling approaches is essential to better understand the secondary metabolite diversity and functionality of *Microcystis*. Training in and advancement of “multi-omic” approaches and modeling will be critical to fully harness these large and complicated data sets.

Field-based studies of diverse and novel *Microcystis* populations that employ *de novo* genome assembly and untargeted MS approaches offer high potential for discovery of novel *Microcystis* secondary metabolites and understanding of the dynamics of *Microcystis* secondary metabolites in real communities as they occur *in situ*. While isolated laboratory cultures are valuable for various analyses, they often fail to represent the breadth and diversity of environmental microbes (172). Efforts to improve this gap, such as the cultivation of over 20 *Microcystis* isolates from a single body of water (133), still suffer from isolation biases and fail to capture the complete diversity of secondary metabolites that *Microcystis* can produce in natural communities. As “meta-omic” studies become more widespread, we see an abundance of previously undescribed and putatively identified BGCs (38, 39, 133, 173) and several new secondary metabolites and congeners (107, 127, 174, 175). Discovery is also highly dependent on *de novo* assembly and untargeted MS approaches as relying on references or standards limits the ability to detect BGCs or metabolites not previously characterized. As we continue to improve analysis pipelines capable of linking genes to compounds, we will need to consider how best to enhance predictive models and machine learning to integrate newly discovered BGCs and metabolites.

## CONCLUSIONS

The extent and diversity of *Microcystis* secondary metabolism is vast and still poorly characterized. Although several *Microcystis* secondary metabolites have been linked to BGCs, the congener diversity, ecological and physiological function, and mechanisms of synthesis remain unresolved for the majority of *Microcystis* secondary metabolites. Future studies should prioritize linking orphan BGCs and metabolites as well as focusing on natural communities as a source for understanding chemical diversity and drivers of synthesis, especially given the diversity of microbial communities and growing evidence that many secondary metabolites are involved in interspecies interactions. This review, like other studies, highlights the need to shift our focus toward other *Microcystis* secondary metabolites besides microcystin as several are abundant in the environment and have toxic properties that may impact food webs or pose threats to public health. Furthermore, the abundance of novel *Microcystis* BGCs and metabolites from both cultured isolates and environmental samples may be sources of novel biotechnological applications or drug discovery. Regardless of motives, the genus *Microcystis* will continue to be a rich source for secondary metabolite research as we are just beginning to understand the range of its biosynthetic potential.
